# The possibilities of a portable low-budget three-dimensional stereophotogrammetry system in neonates: a prospective growth analysis and analysis of accuracy

**DOI:** 10.1186/s13005-018-0168-2

**Published:** 2018-08-03

**Authors:** Lucas M. Ritschl, Maximilian Roth, Andreas M. Fichter, Fabienna Mittermeier, Bettina Kuschel, Klaus-Dietrich Wolff, Florian D. Grill, Denys J. Loeffelbein

**Affiliations:** 1 Department of Oral and Maxillofacial Surgery, Klinikum rechts der Isar, Technische Universität München, Ismaningerstr. 22, D-81675 Munich, Germany; 2 Department of Oral and Maxillofacial Surgery, Helios Klinikum München West, Munich, Germany; 3 Section of Obstetrics, Frauenklinik, Klinikum rechts der Isar, Technische Universität München, Munich, Germany

**Keywords:** Three-dimensional (3D) photogrammetry, Facial analysis

## Abstract

**Background:**

With the technical development, portable three-dimensional (3D) photogrammetry systems are becoming more en vogue because of cost-effectiveness and comparable accuracy to common stationary 3D systems. The purpose of the study was to evaluate the feasibility and accuracy of a low-budget portable system for 3D image acquisition with special regard to the gracile nasal region in neonates. Furthermore, the study aimed to establish a 3D data set of the first 180 days post partum.

**Methods:**

Thirty-three healthy, full-term newborn were enrolled and 3D photographs were prospectively taken monthly with a portable low-budget 3D stereophotogrammetry system (FUEL3D® SCANIFY®) for six months. In the third month, age-matched and corresponding 3D models were acquired by taking an impression of the perinasal area. The resulting plaster models were scanned (3Shape D700, 3Shape® A/S, Denmark). Three examiners analyzed independently 21 defined landmarks of the generated Standard Tessellation Language files with regard to accuracy by using 3dMDvultus™ software. A semi-automatic 3D best-fit analysis of 3D photo and plaster models were performed by using Geomagic® and the Root Mean Squared (RMS) errors were calculated.

**Results:**

Statistically significant changes of midfacial distances and angles with a focus on nasal growth during the first 180 days postpartum could be specified in absolute and relative dimensions. Best-fit analysis in the third month revealed a RMS error of 0.72 ± 0.22 mm with a mean standard deviation of 0.71 ± 0.21 mm.

**Conclusions:**

The analyzed portable 3D stereophotogrammetry system is a feasible methodology with good accuracy, even in newborn. A description of the growth as well as the establishment of a 3D data set was performed. Its implementation for basic documentation for example in cleft patients is possible and might reduce the need for impressions and facilitate the communications with parents and the interdisciplinary team.

## Background

Photo documentation of the face is of fundamental importance for follow-up, communication with patients or parents, illustrative purposes in lectures or medico-legal requirements, and nowadays in surgical planning. Two-dimensional (2D) images have been and are the gold standard for this purpose and can be used to reliably assess phenotypic severity of craniofacial anomalies as stated earlier [1–5]. Considering the drawbacks of conventional 2D photography in picturing three-dimensional (3D) structures and the patient’s exposure to radiation in traditional 3D surface recognition as cephalometry, cone beam scan or computed tomography, non-invasive 3D surface imaging has become more popular in the last decade [6]. It enables non-invasive preoperative illustrative, virtual documentation, planning and simulation [7, 8]. Beside 2D analysis, 3D photography can also be used to analyze facial soft tissue asymmetry [9–11]. More recently, 3D photogrammetry is adapted in the post-operative follow-up of the young patients presenting with cleft lip and palate and/or craniosynostosis [12–15]. But only a minority of studies used portable, hand-held 3D systems including the Artec Eva Scan imaging system, the Vectra H1 system or the M4D Scan system [16–19]. The accuracy is reported to be comparable to common stationary 3D photogrammetry systems and to be sufficient for the most clinical applications [19–21].

The purpose of this study was to analyze a low-budget portable 3D photogrammetry system costing less than 1500 € with a shorter acquisition time compared to the above-mentioned portable systems. Herein a special attention is being paid to accuracy in scanning the nose and the perinasal region. Furthermore, we aimed to evaluate the suitability and feasibility of the application of the system with regard to facial scans in neonates and to describe the growth. Finally the application and integration on neonatal documentation is presented.

## Methods

### Ethical statement and participant acquisition

The study followed the Declaration of Helsinki on medical protocol and ethics and the regional Ethical Review Board of the technical university of Munich approved the study (approval number: 13/16 S). Written participant consent was obtained from the parents and the data collected were pseudonymized. Only healthy full-term Caucasian neonates with unremarkable screening examinations (U1 and U2) were enrolled in collaboration with the Section of obstetrics, Frauenklinik, Klinikum rechts der Isar, Technische Universität München, Germany.

### Facial scans and control model generation

All enrolled neonates were prospectively scanned monthly for a period of the first six postnatal months (180 days), with the first scan to be obtained at the first day postpartum. A variation of ± five days was accepted for the following monthly appointments. Otherwise the taken 3D photo was excluded from analysis. The neonate was also excluded from further analysis, if more than one 3D photo was missed in follow-up. This resulted optimally in six 3D photos per participant in total.

Additionally, a conventional impression of the nose was taken simultaneously to the third facial 3D photo for the generation of a corresponding control model. As previously described, a bluish, semi-transparent a-silicone (Memosil® 2; Kulzer GmbH; Germany) was used for taking the impression [22]. The corresponding plaster models were produced within one hour after impression taking to overcome technical inaccuracy [23]. Following, the plaster models were scanned with a common dental Laser-Scanner (3Shape D700, 3Shape® A/S, Denmark) for Standard Tessellation Language (.STL) file generation and further analysis (see below). The dental Laser-Scanner worked with two integrated cameras (1.3 Megapixels) and a tri-axial joint rotation system so that even deep notches and cavities could be scanned. This quickly provided high resolution scans with an accuracy of 20 μm, according to the manufacturer’s instructions.

### Low-budget portable 3D photogrammetry system

The analyzed 3D photogrammetry system was the FUEL3D® SCANIFY® system scanner, equipped with an adaptable commercially available tablet with a 32-bit Microsoft® Windows® 8 version for data and operational processing. As given in the manufacturer’s instructions [24], the scanner consists of two 3.5 megapixel color-cameras with vertical orientation, three xenon flashlights, three LED searchlights and two release buttons. The integrated hardware delivers a capture speed of approximately 0.1 s. The focus is precalibrated and captures 3D data at a diagonal of circa 40 cm with a resolution of up to 350 μm resulting in up to 375,000 polygons per scan. A white plastic disc with a precisely imprinted black square, surrounding fine lines and one crescent-shaped side marking is needed as a target reference. The target is recognized and the automatic dimensioning is ensured.

The 3D scans were further reprocessed by the corresponding software FUEL3D® Studio 2.2 Professional resulting in. STL and Polygon File Format (.PLY) files.

In both situations, 3D photography and taking nasal impression, the neonates were calm down by the parents and distracted to focus their attention as good as possible. Nevertheless, distraction should not result in facial expressions [25].

### Manual measurement and point-based analysis

**Fig. 1:**
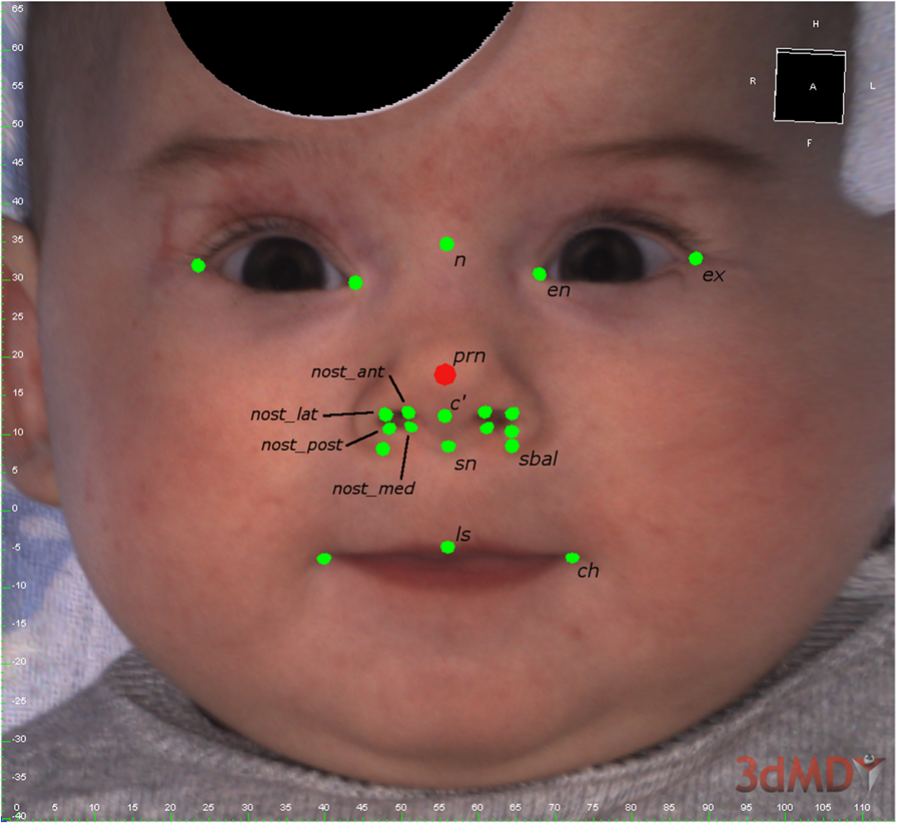
Defined landmarks for point-based, 3D distance and angle analysis of the facial scan (FUEL3D® SCANIFY® system scanner) in frontal perspective using 3dMDVultus software (3dMDvultus™ Software 64-bit 2.4.1.4; 3dMD®; USA) (only one-sided legend and for abbreviations see also Table 1)

Three independent examiners (LMR, FG, MR) performed the manual measurements using 3dMDVultus (3dMDvultus™ Software 64-bit 2.4.1.4; 3dMD®; USA). The analyzed landmarks, as well as the calculated 3D distances and angles are given in Tables 1 and 2 and are illustrated in Fig. 1.

**Table 1:** Definitions of 21 analyzed landmarks as reported earlier by Farkas [44] and extended to the study analysis

Abbreviation	Explanation
*ex*_*r / l*_	Exocanthion _*right / left*_
*n*	Nasion
*en*_*r / l*_	Endocathion _*right / left*_
*prn*	Pronasale
*sbal*_*r / l*_	Subalare _*right / left*_
*sn*	Subnasale
*c’*	Highest point of the columella
*ls*	Labiale superius
*ch*_*r / l*_	Cheilion _*right / left*_
*nost_post*_*r / l*_	posterior border of nostril _*right / left*_
*nost_ant*_*r / l*_	anterior border of nostril _*right / left*_
*nost_lat*_*r / l*_	lateral border of nostril _*right / left*_
*nost_med*_*r / l*_	medial border of nostril _*right / left*_

**Table 2:** Definitions of 15 analyzed 3D distances and 6 angles

	Abbreviation	Explanation
3D distances	*n-sn*	nasal height
	*en*_*r*_ *-en*_*l*_	intercanthal distance
	*(ex-en)*_*r / l*_	eye width _*right / left*_
	*sbal*_*r*_ *-sbal*_*l*_	subalar width
	*sn-nt*	nasal depth
	*(en-sbal)*_*r / l*_	lateral height of the nose _*right / left*_
	*c’-sn*	length of the columella
	*sn-ls*	lengt of the upper lip
	*ch*_*r*_ *-ch*_*l*_	oral width
	*(en-ch)*_*r / l*_	lateral height of the midface _*right / left*_
	*(nost_post - nost_ant)*_*r / l*_	length of the nostril _right / left_
	*(nost_lat - nost_med)*_*r / l*_	width of the nostril _right / left_
3D angles	*n - nt - sn*	nasal tip angle
	*ls - sn - nt*	subnasal angle
	*ls - sn - c’*	approximated nasolabial angle
	*sbal*_*r*_ *- nt - sbal*_*l*_	anterior angle of the nasal base triangle defined by the mentioned points
	*sbal*_*r*_ *- sn - sbal*_*l*_	anterior angle of the nasal base triangle defined by the mentioned points
	*en*_*r*_ *- n - en*_*l*_	anterior angle of the nasal root triangle defined by the mentioned points

In a first step, a mean point was calculated for each landmark and dataset and was used as a 3D analysis of reliability. Three dimensional distances and angles were automatically calculated based on the mean points for each dataset.

Finally, growth analysis in relation to the participant’s age (days [d]) was conducted and visualized in graphs for each participant. Absolute information of growth is given in millimeters [mm] or degrees [°], the relative information of changes relate to the calculated differences between the measurements at *t* = 1d and t = x (i.e. the investigated point in time) and is given in percent [%].

### Superimposition and surface-based, semi-automatic analysis

In order to evaluate the accuracy of the described 3D facial scanner, a surface-based analysis was performed using Geomagic (Geomagic® Control, Version 2014, USA). The integrated best-fit algorithm semi-automatically superimposed the control model and its corresponding facial scans. Second, the orthogonal distances between the surface points of the cast to the corresponding surface of the facial scan were registered and the Root Mean Squared (RMS) errors were calculated automatically. The resulting statistical reports of each model pair were subsequently combined in a single table for further analysis.

### Statistical analyses

The data were analyzed with IBM® SPSS® 25.0 for Mac (IBM Corp, Armonk, NY, USA). Figures were generated with Excel® (Microsoft Excel® 15.39 for Mac, Microsoft Corp., Redmond, WA, USA).

The given *p*-values were calculated by two-tailed tests including Bonferroni correction and are subject to a global significance level of 0.05.

## Results

### Study collective

The study collective consisted initially of 34 Caucasian neonates of whom 33 were observed and included for further analysis. The parents of one child ended the participation because of lack of time after two scans had already been taken. These two scans were excluded from our analysis due to the insufficient follow-up. Two parents did not allow taking an impression of their enrolled child. This resulted in 31 corresponding plaster models in total for the surface-based analysis and comparison between 3D photo and plaster model. Final gender distribution was homogenous including 16 males and 17 females.

### Growth analysis

**Fig. 2:**
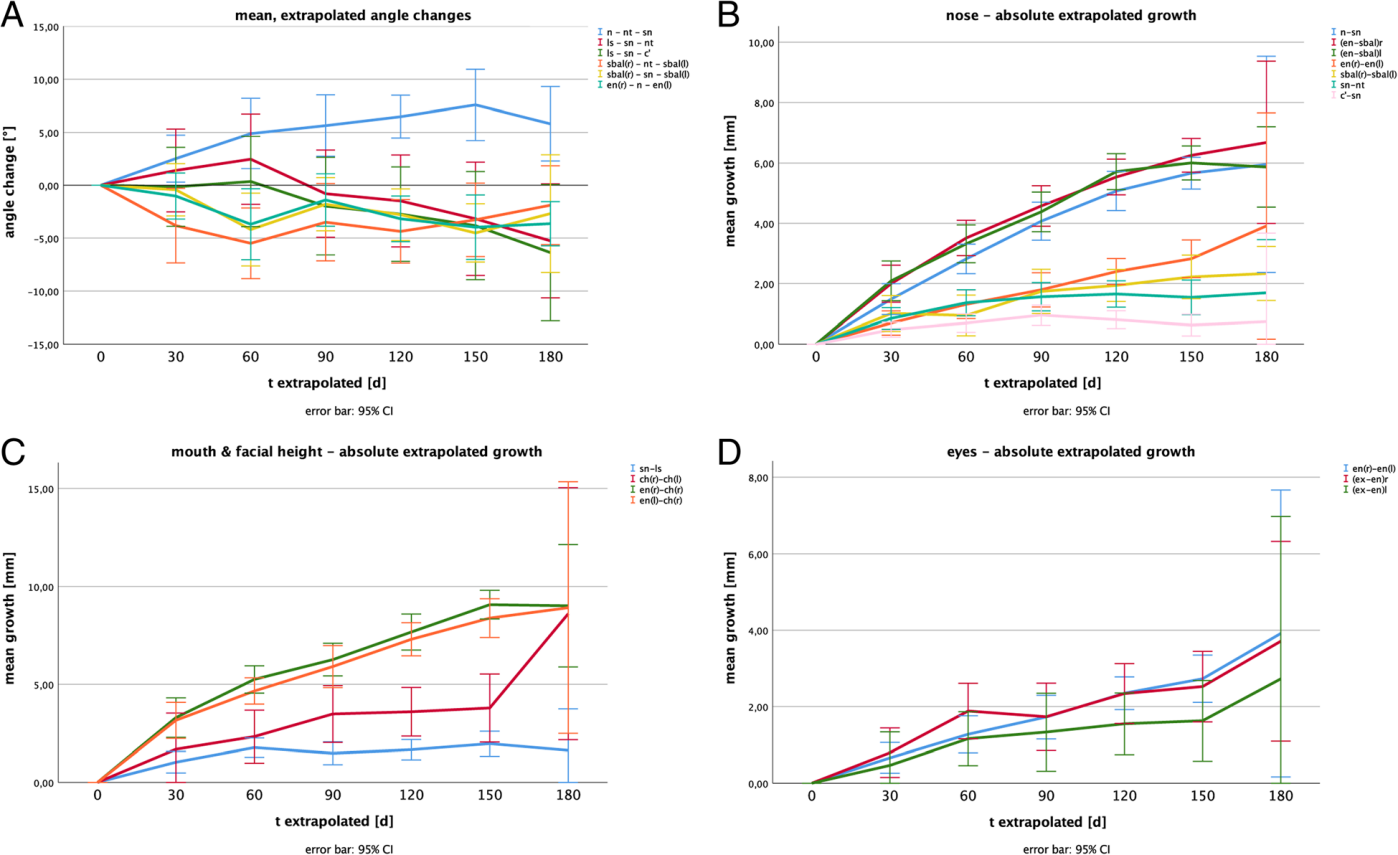
Illustration of 3D changes based on the extrapolated data within a 180 days observation period: **a** Absolute, extrapolated growth of nasal parameters; **b** Mean, extrapolated changes of nasal angles; **c** Absolute, extrapolated growth of oral and midfacial parameters and (**d**) Absolute, extrapolated growth of ocular parameters (the error bars indicate 95% confidence intervals)

Mean 3D distances and angles, as well as mean absolute and relative growths are given in Tables 3 and 4. Figure 2 (A-D) illustrates the dimensional changes based on the extrapolated data. The error bars indicate 95% confidence intervals.

**Table 3:** Mean extrapolated changes of 3D distances within the first postnatal 180 days

	Mean 3D distances t extrapolated [d]																			
	t = 1d (a)		30 (b)			60 (c)			90 (d)			120 (e)			150 (f)			180 (g)		
	Mean [mm]	SE [mm]	Mean [mm]	SE [mm]	%	Mean [mm]	SE [mm]	%	Mean [mm]	SE [mm]	%	Mean [mm]	SE [mm]	%	Mean [mm]	SE [mm]	%	Mean [mm]	SE [mm]	%
*n-sn*	20.82	0.30	1.49 ^a^	0.25	7.1	2.85 ^a,b^	0.24	13.7	4.07 ^a,b,c^	0.31	19.5	5.07 ^a,b,c^	0.32	24.4	5.67 ^a,b,c,d^	0.26	27.2	5.95 ^a,b,c^	0.83	28.6
intercanthal distance	22.09	0.30	0.70	0.20	3.2	1.25 ^a^	0.23	5.7	1.79 ^a,b^	0.28	8.1	2.40 ^a,b,c^	0.21	10.9	2.82 ^a,b,c^	0.30	12.8	3.91 ^a,b,c^	0.87	17.7
*ex-en*_*r*_	21.58	0.36	0.81	0.31	3.8	1.82 ^a^	0.35	8.4	1.67 ^a^	0.42	7.7	2.36 ^a,b^	0.38	10.9	2.51 ^a,.b^	0.43	11.6	3.71 ^a^	0.61	17.2
*ex-en*_*l*_	21.73	0.40	0.47	0.43	2.1	1.16	0.35	5.3	1.33	0.49	6.1	1.55	0.40	7.1	1.63	0.51	7.5	2.72	0.99	12.5
*sbal*_*r*_ *- sbal*_*l*_	16.11	0.28	1.01	0.29	6.3	1.04	0.33	6.4	1.74 ^a^	0.36	10.8	1.94 ^a^	0.26	12.0	2.22 ^a^	0.35	13.8	2.34	0.21	14.5
*sn-nt*	9.32	0.22	0.85 ^a^	0.18	9.1	1.39 ^a^	0.21	14.9	1.56 ^a^	0.23	16.8	1.66 ^a^	0.21	17.8	1.54 ^a^	0.28	16.6	1.70	0.41	18.2
*en-sbal*_*r*_	18.32	0.31	2.00 ^a^	0.30	10.9	3.54 ^a,b^	0.28	19.3	4.58 ^a,b^	0.33	25.0	5.54 ^a,b,c^	0.29	30.2	6.26 ^a,b,c,d^	0.27	34.2	6.68 ^a,b,c^	0.62	36.5
*en-sbal*_*l*_	18.41	0.32	2.09 ^a^	0.32	11.4	3.30 ^a,b^	0.30	17.9	4.38 ^a,b^	0.32	23.8	5.72 ^a,b,c,d^	0.29	31.0	6.00 ^a,b,c,d^	0.27	32.6	5.87 ^a,b^	0.31	31.9
*c’-sn*	4.14	0.12	0.48	0.12	11.6	0.69 ^a^	0.15	16.8	0.96 ^a^	0.16	23.1	0.81 ^a^	0.14	19.5	0.63 ^a^	0.17	15.2	0.74	0.68	17.9
*sn-ls*	9.17	0.30	1.11 ^a^	0.25	12.1	1.73 ^a^	0.23	18.9	1.51 ^a^	0.26	16.4	1.69 ^a^	0.24	18.4	1.97 ^a^	0.30	21.5	1.62	0.49	17.7
*ch*_*r*_ *- ch*_*l*_	27.75	0.46	1.68	0.90	6.1	2.33	0.66	8.4	3.48 ^a^	0.70	12.5	3.59 ^a^	0.61	13.0	3.79 ^a^	0.84	13.6	8.60 ^a,b^	1.50	31.0
*en-ch*_*r*_	31.08	0.43	3.37 ^a^	0.46	10.8	5.27 ^a,b^	0.33	17.0	6.27 ^a,b^	0.39	20.2	7.76 ^a,b,c^	0.45	25.0	9.07 ^a,b,c,d^	0.35	29.2	9.01 ^a,b,c^	0.73	29.0
*en-ch*_*l*_	31.32	0.41	3.16 ^a^	0.44	10.1	4.66 ^a^	0.33	14.9	5.91 ^a,b^	0.52	18.9	7.30 ^a,b,c^	0.42	23.3	8.38 ^a,b,c,d^	0.48	26.8	8.92 ^a,b,c^	1.49	28.5
*nost_post - nost_ant*_*r*_	4.91	0.11	0.49	0.12	10.0	0.93 ^a^	0.16	19.0	1.24 ^a,b^	0.14	25.3	1.08 ^a,b^	0.12	22.0	1.21 ^a,b^	0.18	24.6	1.53 ^a^	0.17	31.1
*nost_lat - nost_med*_*r*_	4.06	0.08	0.38	0.08	9.3	0.55 ^a^	0.11	13.6	0.65 ^a^	0.10	16.0	0.80 ^a,b^	0.11	19.7	0.61 ^a^	0.12	15.1	0.51	0.29	12.5
*nost_post - nost_ant*_*l*_	5.10	0.12	0.29	0.11	5.6	0.92 ^a,b^	0.18	18.1	1.08 ^a,b^	0.15	21.2	0.99 ^a,b^	0.14	19.4	0.97 ^a,b^	0.17	19.0	0.94	0.28	18.5
*nost_lat - nost_med*_*l*_	3.66	0.08	0.42	0.10	11.4	0.55 ^a^	0.11	15.0	0.70 ^a^	0.11	19.1	0.82 ^a^	0.13	22.4	0.93 ^a,b^	0.13	25.4	0.82	0.19	22.3

_*l*_ left, _*r*_ right

^a / b / c / d^significant change in relation to column a / b / c / d with *p* < 0.05; unidirectional link only to prior values

**Table 4:** Mean extrapolated changes of angles within the first postnatal 180 days

	Mean angles t extrapolated [d]																			
	t = 1d (a)		30 (b)			60 (c)			90 (d)			120 (e)			150 (f)			180 (g)		
	Mean [–]	SE [–]	Mean [–]	SE [–]	%	Mean [–]	SE [–]	%	Mean [–]	SE [–]	%	Mean [–]	SE [–]	%	Mean [–]	SE [–]	%	Mean [–]	SE [–]	%
*n – nt - sn*	116.35	1.13	2.32	1.10	2.0	4.51	1.54	3.9	5.10	1.45	4.4	5.92 ^a^	1.02	5.1	7.64 ^a^	1.56	6.6	5.81	1.49	5.0
*ls – sn - nt*	*154.42*	*1.64*	*1.40*	*1.90*	*0.9*	*2.46*	*2.09*	*1.6*	*−0.80*	*2.00*	*−0.5*	*−1.48*	*2.14*	*−1.0*	*−3.17*	*2.60*	*−2.1*	*−5.26*	*2.28*	*−3.4*
*ls – sn – c’*	138.66	1.57	−0.15	1.81	−0.1	0.35	2.09	0.3	−1.97	2.24	−1.4	−2.73	2.19	−2.0	−3.82	2.47	−2.8	−6.37	2.72	−4.6
*sbal*_*r*_ *– nt - sbal*_*l*_	73.48	1.61	−2.31	1.55	−3.1	−5.04	1.57	−6.9	−3.23	1.73	−4.4	−4.03	1.44	−5.5	−3.73	1.68	−5.1	−1.88	1.57	−2.6
*sbal*_*r*_ *– sn - sbal*_*l*_	142.37	1.16	1.16	1.34	0.8	− 3.67	1.62	−2.6	−1.52	1.21	−1.1	−2.04	1.35	−1.4	−4.67 ^b^	1.29	−3.3	−2.68	2.35	−1.9
*en*_*r*_ *– n - en*_*l*_	121.42	1.03	−0.96	1.00	−0.8	−3.74	1.54	−3.1	−1.54	1.17	−1.3	−3.11	1.05	−2.6	−4.08	1.42	−3.4	−3.64	0.88	−3.0

^a / b^ significant change in relation to column a / b with *p* < 0.05; unidirectional link only to prior values

### *Nasal angles* (Fig. 2a)

The sagittal nasal tip angle (*n - nt - sn*) showed a statistical significant increase of 5.92 ± 1.02 ° (5.1%) 120 days postpartum.

Also the posterior nasal base angle (*sbal*_*r*_ *- sn - sbal*_*l*_) was found to significantly decrease by 4.67 ± 1.29 ° (3.3%) after 150 days.

All other measured angles did not reveal any statistically significant changes, even though a slight trend towards a change of approximately − 4° after 150 days was found for the nasiolabial angle (*ls - sn - c’*), the anterior nasal base angle (*sbal*_*r*_ *- nt - sbal*_*l*_) as well as for the intercanthal angle (*en*_*r*_ *- n - en*_*l*_).

### Nasal parameters (Fig. 2b)

The nose was observed to grow nearly linear in its longitudinal axis (*n - sn*) with a statistical significant increase of approximately 6–7% per month during the first 90 days. Subsequent the growth rate decreased resulting in a total significant growth of 5.67 ± 0.26 mm (27.2%) after 150 days. After 180 days an increased standard error of the mean value (SE) of ±0.83 mm needs to be mentioned. The lateral nasal height (*en - sbal*) showed similar growth rates with slightly higher growth rates during the first 60 days (~ 9% per month) resulting in a total growth of 6.26 ± 0.27 mm (34.2%) on the right, respectively 6.00 ± 0.27 mm (32.6%) on the left side after 150 days. The measurements at *t* = 180 days postpartum also showed higher variances, resulting in non-significant changes in relation to the measurements at *t* = 150 days postpartum (Table 3).

In the sagittal axis, the nasal depth (*sn - nt*) was found to grow rapidly during the first 60 days with 1.39 ± 0.21 mm (14.9%) after 60 days, then leveling at an absolute growth of approximately 1.6 mm (~ 17%) in relation to the starting point until the end of the observation period. Significant differences between the different measurements were only found in relation to t = 1 day. The columella was found to grow similarly with a total increase in length of 0.81 ± 0.14 mm (19.5%) after 120 days. A first significant change was found for *t* = 60 days in relation to the starting point, the following measurements did not show other statistically significant changes in between the measurements.

The basal nasal width (*sbal*_*r*_ *- sbal*_*l*_) seemed to increase rather slowly with a first statistically significant growth after 90 days (1.74 ± 0.36; 10.8%) leveling at approximately 2 mm (~ 12%). All other intervals did not show more statistically significant differences than in relation to the first day postpartum.

### Midfacial parameters (Fig. 2c)

The lateral midfacial height (*en - ch*) showed an increase according to its central pendant of the nasal height with a steep increase of slightly more than 10% during the first 30 days, slowly flattening and reaching a growth of approximately 20% after 90 days, and approximately 27% after 150 days. Except for the measurements after 60 days the monthly intervals showed statistically significant differences to their previous values. Again the measurements at *t* = 180 days postpartum showed higher variance.

The upper lip (*sn - ls*) lengthened by approximately 1.7 mm in total (18.0%) with a significant growth of 1.11 ± 0.25 mm (12.1%) during the first 30 days postpartum. The following intervals did not show other significant differences.

The oral width increased by 3.59 ± 0.61 mm (13.0%) during the first 120 days. A first statistically significant difference was found for *t* = 90 days postpartum. Comparisons of other intervals did not reveal more significant findings. The oral width at t = 180 days postpartum was found to show the highest spread of all values (growth of 8.60 ± 1.50 mm).

### Ocular parameters (Fig. 2d)

Eye width and intercanthal distance were found to grow nearly linear with a significant increase of the intercanthal distance of 2.82 ± 0.30 mm (12.8%) after 150 days, and a significant growth of the right eye width of 2.51 ± 0.43 mm (11.6%) after 150 days. For the left eye width, no significant growth could be registrated even though the measured distances were similar.

### Surface-based accuracy analysis

Four. STL files of the corresponding plaster models were excluded because of heavy insufficiency of the impression. This resulted in 27 model pairs of age-matched corresponding. STL files (3D photo vs. plaster model).

Regarding all model pairs, the mean Root Mean Squared error (RMS error) was 0.72 ± 0.22 mm between the superimposed surfaces.

The mean upper deviation was 0.51 ± 0.14 mm, the mean lower deviation was − 0.58 ± 0.22 mm resulting in a mean standard deviation of 0.71 ± 0.21 mm.

**Fig. 3:**
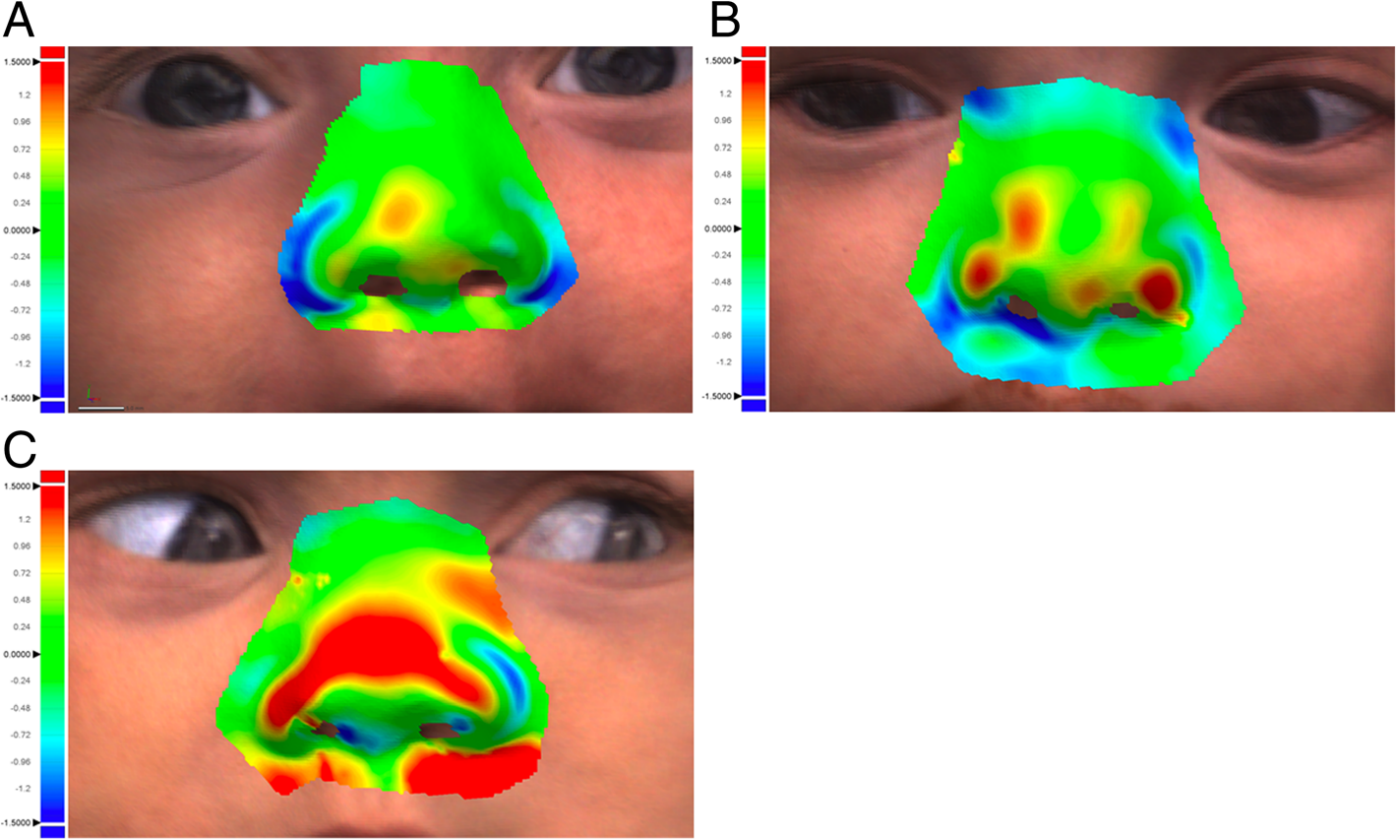
The surface-based accuracy analysis with color-coded surface mismatch error using Geomagic (Geomagic® Control, Version 2014, USA) with a good (**a**), intermediate (**b**) and insufficient (**c**) result between calculated surface deviation of the corresponding .STL files 3D photo using the FUEL3D® SCANIFY® system scanner versus plaster model

Anatomical areas with rather severe curvature, overlapping surfaces or tangential point of view were found to show rather high deviation of the superimposed surface datasets (e.g. the posterolateral alar sidewalls, parts of the nostrils or the lower parts of the nasal tip). Representatively these areas are illustrated in Fig. 3.

## Discussion

**Fig. 4:**
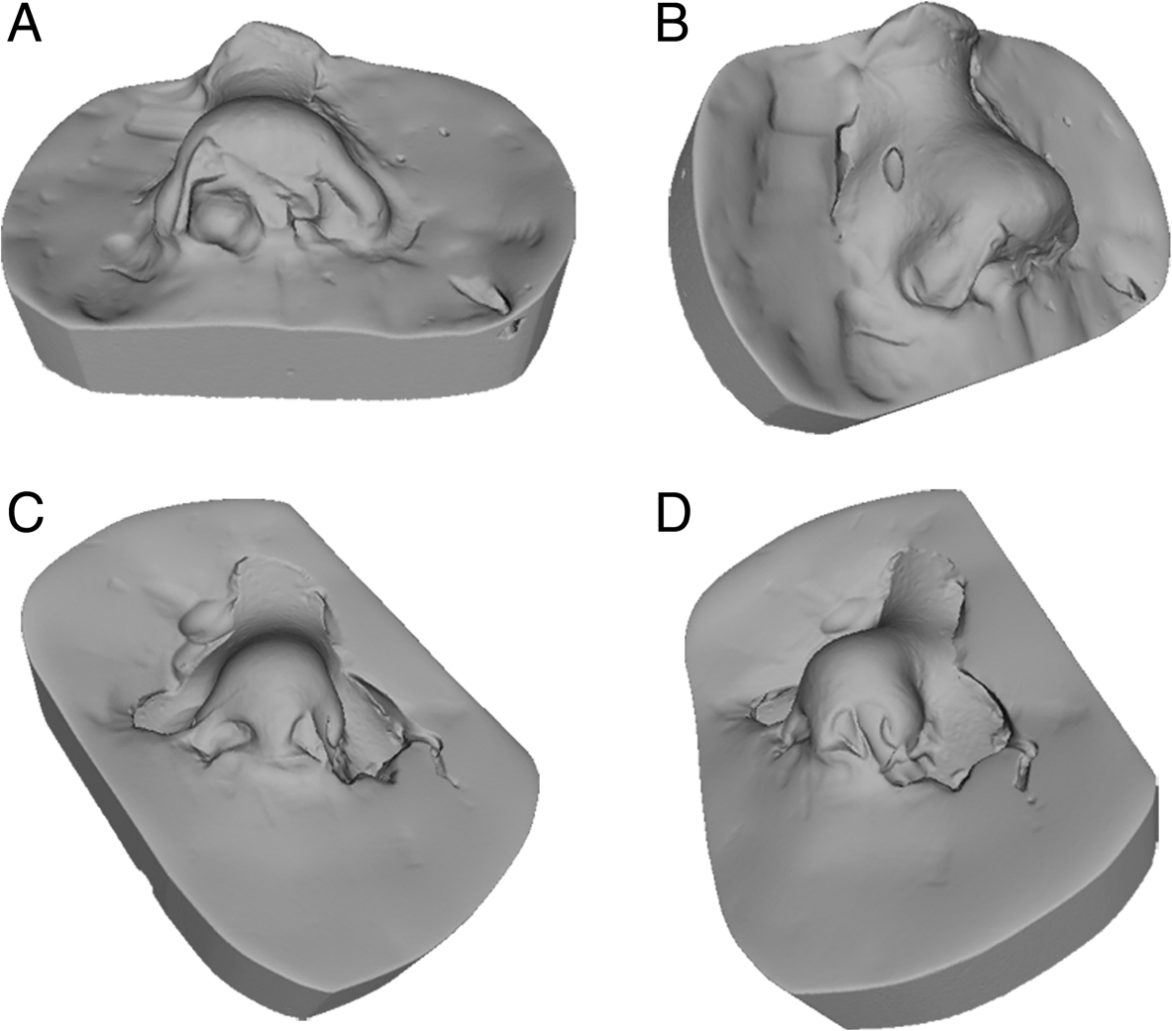
Two exemplary cases of excluded plaster models because of low quality of impression and consecutive plaster model generation. As seen in caudal view (**a** and **c**) and in latero-caudal view alar (**b** and **d**) nose regions remained demanding. Further, correct and sufficient impressions of the nostrils are also difficult

Conventional photo documentation of the face of neonates and children as well as of adults is well documented [2, 5]. Taking impressions with different techniques and materials for detailed, reliable, safe and non-invasive 3D registration is the gold standard procedure and is also well described in the literature [22]. But preliminary, the authors want to highlight a relevant issue for the further interpretation of results. Direct methods which need contact to the patient’s soft tissue to acquire 3D models, as seen in classical impression taking, might result in measurement errors caused by modification of the soft tissue, facial movement or grimacing [26, 27]. Especially involuntary movements during taking impressions were relevant in our study and is also seen in our results. In four cases we could not include the plaster models, because of too low quality of the impressions due to movement of the children (Fig. 4). Further, impression materials and techniques, and plaster model generation might result in previously described inaccuracies in cast production [28]. For the mentioned reasons we preferred to use A-silicone as material and produced the plaster model within 1 hour [22]. Additionally, scanning procedure of dental casts is also associated with inaccuracies, which cannot be neglected. In our study we used a common dental scanner with an accuracy of 20 μm. In summary, the indirect way of surface information acquisition is always associated with possible theoretical errors [23, 29]. For that reason, the. STL file of the 3D photography was rather compared to the resulting. STL file of the plaster model than to the theoretical “truth”. On the other hand, studies comparing only 3D photos compare only the “theoretical truth” with all the inaccuracies of the used systems [21].

In clinical translation, especially in neonates and young children, taking impressions remains challenging and therefore clinically feasible and reliable alternatives are wanted for sufficient registration, follow-up and treatment planning. For the purpose of non-invasive extraoral registration, 3D photography is becoming more popular and several studies imply a better assessment of cleft-related deformities than in conventional 2D photography [30, 31]. The analyzed facial scanner (FUEL3D® SCANIFY® system scanner) had a user-friendly interface and the associated software was easy to use. Regarding the acquisition costs, the FUEL3D® SCANIFY® system scanner is a low-cost 3D surface scanner.

In comparison to other portable systems, our analyzed device had a comparable, acceptable, low RMS error of 0.72 ± 0.22 mm [16, 20, 21]. Even in comparison to reported results of stationary photogrammetry systems like 3dMDface or Vectra, our RMS error seems to be acceptable and keeps up with these systems [32–34]. Fan et al. also analyzed a low-cost 3D Scanner ($ 400). But in contrast to our good results, they reported an overall Target Resulting Error of 2.5 ± 0.31 mm [35]. This is worse than our results and is on the border for clinical relevance, since deviations larger than 2 mm are considered unreliable.

Our results are in three ways interesting and worthwhile to follow. First, RMS error is comparable to above-mentioned systems, but the analyzed FUEL3D® SCANIFY® system scanner costs only about 1500 €. This is far less than a 3dMD system or other described handheld solutions. Three-dimensional photo documentation could be affordable even for smaller clinics/institutions or in private practice with this system. Second, deviation of more than 2 mm is considered to be unreliable and the assessment of areas with high curvatures and shadows can be difficult [19, 36]. In contrast to other studies, the corresponding models in the third month of life (3D photo vs. plaster model) only captured the probably most demanding anatomical area of the face, the perinasal region. Nevertheless, our results remained good. And last, the evaluated 3D system is the very fast capture speed of approximately 0.1 s per picture. The resulting 3D photo is a single 3D photograph and is not based on a sequence of 3D pictures as seen in the Artec Eva scanner for example [16]. This enables an unaltered picture, even in cases of involuntary facial movements during surface recognition or scanning as stated earlier [37]. Nevertheless, even in our collective the most varying landmarks and corresponding 3D distance were cheilion right/left and the oral width at 180 days postpartum which was associated with the highest spread of all values (growth of 8.60 ± 1.50 mm). Mouth movements of the newborn might explain these findings. An already described solution is the capturing of a video sequence, as done with 3dMDface or Artec Eva system for example. But herein, the volume of data increases significantly and detailed surface data could get lost during post-processing procedures [16]. On the other hand, anatomical areas with rather severe curvature or overlapping surfaces were found to show higher deviations in our best-fit analysis of the superimposed surfaces. This might be due to the vertical location of the two integrated cameras in the evaluated system and their rather small distance to each other compared to stationary scanners. To optimize the results, a third camera may improve the accuracy.

We analyzed neonates within the first 180 days of life. As mentioned earlier, facial surface registration is challenging in this cohort. This is not the first study of neonates/newborn using 3D systems and scanner. But most 3D photogrammetry systems were only used to perform a descriptive analysis of the post-operative result for example after primary lip repair in children with cleft lip and palate or in patients with craniosynostosis [11–14]. Our study analyzed the growth of the perinasal region in healthy unaffected newborn. These results can be used as a 3D data set of the optimal perinasal region and its development. As described by others, the major deformity for example in patients with cleft lip and palate results in the nose and secondarily in the midfacial region [38]. For this purpose the knowledge of healthy growth is important and in the age of virtualization additional information to the conventional 2D photography is needed [39]. 3D photography is nowadays possible with an accurate quality and can be used for documentation, even with portable solutions. But according to Kuijpers et al. no data have yet been able to show that 3D methods are more informative than conventional 2D methods [40]. Chou et al. fully agreed with us in seeing the benefits of consecutive integration of 3D photo documentation in children. They documented prospectively the growth of neonates with unilateral cleft lip and palate during nasoalveolar molding (NAM) therapy with weekly photographs [15]. No impressions were needed. According to our experience with NAM [41], the therapy can be easier adopted, when 3D models are present. This eases the follow-up and can also be used to show the instant therapeutic effect to the parents, which might further improve the mandatory compliance for this therapy.

In a next step we plan to integrate the extraoral three-dimensional data to further improve our final goal, the automated production of NAM plates using the CAD/CAM technique as described earlier [42, 43]. Further we will continue the observation of our included children in order to generate a systematic reference pool for the growing face, based on 3D photographical data acquisition.

### Limitations

One limitation of the study is, that only one 3D system or scanner was used. But according to the literature, plaster models of good impressions remain the gold standard. Classical anthropometry with real-time measurement would not have been feasible and a surface analysis would have been impossible in neonates. Another limitation might the fact that only one corresponding plaster model was used for comparison. But the regional ethical review board of the technical university of Munich only approved one perinasal impression in the healthy neonates at the age of 3 months. Last, the RMS error represents only the difference between the compared. STL files of the resulting scans and not necessarily between the real perinasal region and the 3D photo.

## Conclusions

The low-budget FUEL3D® SCANIFY® system scanner seems to have acceptable accuracy compared to other established systems and is feasible to use despite its technical simplicity. Severe curvature and overlapping surfaces are associated with greater inaccuracy because of its vertical orientation of the integrated two cameras. 3D growth analyses were easily possible and enabled us to establish a optimal 3D data set of the developing midfacial region. The cost-effectiveness of the system is suitable for most common applications with regard to facial scans in neonates.

## Abbreviations

.PLY file: Polygon File Format file
.STL file: Standard Tessellation Language file
2D: two-dimensional
3D: three-dimensional
RMS error: Root Mean Squared error
